# Left Ventricular Noncompaction in Advanced Heart Failure With an Anomalous Coronary Artery: A Case Report

**DOI:** 10.7759/cureus.80015

**Published:** 2025-03-04

**Authors:** Khaleel Quasem, Michelle Carrasquel, Jordan Felice, Britni Smith, Dania Baraka, Karam Khasawneh, Adam Quasem, Majid Mughal

**Affiliations:** 1 Internal Medicine, McLaren Greater Lansing, Lansing, USA; 2 Cardiology, McLaren Greater Lansing, Lansing, USA; 3 Internal Medicine, Central Michigan University College of Medicine, Mount Pleasant, USA; 4 Internal Medicine, Michigan State University College of Human Medicine, East Lansing, USA

**Keywords:** adult congenital heart disease (achd), cardiology, cardiomyopathy, congenital heart disease, left ventricular non compaction

## Abstract

Left ventricular noncompaction (LVNC) involves abnormal development of the heart muscle, where the inner layer remains excessively trabeculated instead of compacting properly. Traditionally considered congenital, increasing reports of nonfamilial or sporadic LVNC suggest that adverse myocardial remodeling, such as from volume overload or preexisting cardiomyopathy, may contribute to its development. We present the case of a 77-year-old male with chronic atrial fibrillation and nonischemic cardiomyopathy who was found to have severe LVNC, identified on echocardiography and cardiac MRI using a noncompacted-to-compacted myocardial thickness ratio >2.3 at end-systole. Severe left ventricular dysfunction (ejection fraction (EF) <20%) was confirmed, and angiography revealed nonobstructive coronary disease with an anomalous left circumflex artery - an uncommon co-occurrence that underscores the importance of comprehensive imaging. The late presentation of LVNC in an older adult without a familial history expands the recognized demographic and suggests that structural and hemodynamic stressors may play a role in its development. Management included pharmacologic rate control for atrial fibrillation, electrical cardioversion, and guideline-directed therapy for heart failure with reduced EF, ultimately leading to implantable cardioverter-defibrillator placement for primary prevention of sudden cardiac death. Following these interventions, the patient showed modest improvement in functional status and symptoms and remains under close follow-up for device surveillance and serial imaging. This case broadens our understanding of LVNC by highlighting its potential for late onset, the necessity of multimodality imaging to detect coexisting anomalies, and the importance of a comprehensive treatment approach to optimize outcomes in older adults.

## Introduction

Left ventricular noncompaction (LVNC) is a rare cardiomyopathy characterized by excessive trabeculation of the myocardium, resulting from incomplete compaction of normal myocardial fibers [1]. This process creates a thick, noncompacted endocardial layer over a thinner compacted epicardium. While LVNC was traditionally considered a congenital disorder, emerging evidence suggests it can also be acquired, indicating a multifactorial etiology [2,3]. Myocardial remodeling due to volume overload or progressive changes in other cardiomyopathies has been proposed as a potential mechanism for acquired LVNC. This abnormal myocardial structure can lead to significant ventricular dysfunction, increasing the risk of heart failure, arrhythmias, and thromboembolic events - underscoring the importance of early detection and appropriate management [4].

LVNC presents along a broad clinical spectrum. Some individuals remain asymptomatic and receive a diagnosis incidentally, while others develop severe cardiac complications [1,2]. In some cases, arrhythmias serve as the initial clinical manifestation, preceding the onset of symptomatic heart failure or thromboembolic complications. These arrhythmias, ranging from atrial fibrillation to potentially life-threatening ventricular tachyarrhythmias, are thought to arise from structural irregularities that disrupt normal electrical conduction. Diagnosis relies on echocardiography and cardiac MRI [3,5]; however, distinguishing LVNC from other cardiomyopathies, such as dilated or hypertrophic cardiomyopathy with deep trabeculations, remains challenging due to overlapping structural and functional characteristics. Careful imaging interpretation, combined with clinical context, is essential for an accurate diagnosis.

Several factors influence the progression of LVNC to overt heart failure or significant arrhythmias, including the extent of noncompaction, left ventricular systolic dysfunction, underlying comorbidities (e.g., hypertension and coronary artery disease), and early manifestations of arrhythmias. Management primarily focuses on symptom relief, arrhythmia prevention, and thromboembolic risk reduction through anticoagulation. In advanced cases, implantable cardioverter-defibrillators (ICDs) or even heart transplantation may be necessary. Despite medical advancements, LVNC remains underrecognized and poorly understood, limiting the availability of robust data to refine management strategies.

Here, we present a unique case of LVNC that highlights the role of advanced diagnostic imaging, outlines the clinical course, and details the therapeutic approach, while also illustrating the rarity of LVNC coinciding with an anomalous coronary artery. This co-occurrence is clinically significant, as it underscores the potential for coexisting structural cardiac anomalies that may alter the presentation and management of LVNC. Comprehensive imaging was pivotal in detecting both the noncompacted myocardial segments and the anomalous coronary origin, guiding the subsequent treatment plan.

## Case presentation

A 77-year-old male with a history of hypertension, paroxysmal atrial fibrillation, nonischemic cardiomyopathy (diagnosed approximately one year earlier but not extensively evaluated), and hyperlipidemia presented to the ED after several weeks of progressively worsening dyspnea. On arrival, he was tachycardic (heart rate: 111 bpm) and hypotensive (blood pressure: 92/82 mmHg), with a respiratory rate at the upper limit of normal (20 breaths/min) and an oxygen saturation of 93% on room air (Table 1).

**Table 1:** Initial presenting vital signs The vital signs indicate tachycardia, tachypnea, and hypotension.

Vital sign	Value	Reference range
Heart rate	111 bpm	60-100 bpm
Respiratory rate	20 breaths/min	12-20 breaths/min
Blood pressure	92/82 mmHg	90/60-120/80 mmHg
Oxygen saturation	93%	96-100%

An outpatient echocardiogram performed the previous year had shown a significantly dilated left ventricle with global hypokinesis, an estimated ejection fraction (EF) of 20-25%, and notably prominent trabeculations in the mid-lateral and apical walls. However, at that time, the possibility of LVNC had not been definitively investigated.

**Figure 1:**
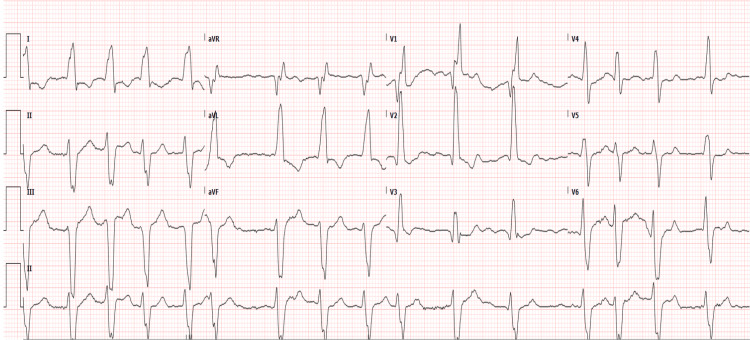
12-lead EKG A 12-lead EKG demonstrates atrial fibrillation with a ventricular rate of 95 bpm. The QRS complex is widened (165 ms), indicative of a likely right bundle branch block. QTc is within normal limits, with left axis deviation. No evidence of ST elevation or depression is observed.

Upon presentation to the ED, a 12-lead EKG showed atrial fibrillation with a widened QRS complex (165 ms), consistent with right bundle branch block (Figure 1). Laboratory studies revealed mild leukocytosis (WBC 11.78 × 10⁹/uL), an elevated NT-proBNP level (4,362 pg/mL), and an increased high-sensitivity troponin (59 ng/L), suggestive of decompensated heart failure and ongoing myocardial stress (Table 2). Hemoglobin was 16.3 g/dL, and hematocrit was 51.2%, effectively ruling out true anemia. These findings, along with his hemodynamic instability, indicated an acute exacerbation of his chronic cardiac condition.

**Table 2:** Initial laboratory results The laboratory results are notable for elevated hs-cTN and NT-proBNP, as well as anemia. ALP, alkaline phosphatase; ALT, alanine aminotransferase; AST, aspartate aminotransferase; BUN, blood urea nitrogen; Cr, creatinine; eGFR, estimated glomerular filtration rate; Hct, hematocrit; Hgb, hemoglobin; hs-cTN, high-sensitivity cardiac troponin; NT-proBNP, N-terminal pro B-type natriuretic peptide

Category	Test	Value	Reference range
Hematology	WBC	11.78 × 10⁹/uL	4.0-11.0 × 10⁹/uL
	Hgb	16.3 g/dL	13.5-17.5 g/dL
	Hct	51.20%	41-53%
	Platelets	202 × 10³/µL	150-450 × 10³/µL
Electrolytes	Sodium	142 mmol/L	135-145 mmol/L
	Potassium	4.4 mmol/L	3.5-5.0 mmol/L
	Chloride	107 mmol/L	96-106 mmol/L
	Calcium	9.7 mg/dL	8.5-10.5 mg/dL
	Magnesium	2.3 mg/dL	1.8-2.5 mg/dL
	CO₂	22.4 mmol/L	22-29 mmol/L
	Anion gap	12.6 mmol/L	7-16 mmol/L
Renal function	BUN	30.7 mg/dL	6-20 mg/dL
	Creatinine	1.9 mg/dL	0.7-1.3 mg/dL
	eGFR	36 mL/min/1.73 m²	>90 mL/min/1.73 m²
Liver function	ALT	79 U/L	7-56 U/L
	AST	41 U/L	10–40 U/L
	ALP	109 U/L	44-147 U/L
	Bilirubin total	0.7 mg/dL	0.1–1.2 mg/dL
Cardiac function	NT-proBNP	4,362 pg/mL	<125 pg/mL
	hs-cTN	59 ng/L	<20 ng/L

**Figure 2:**
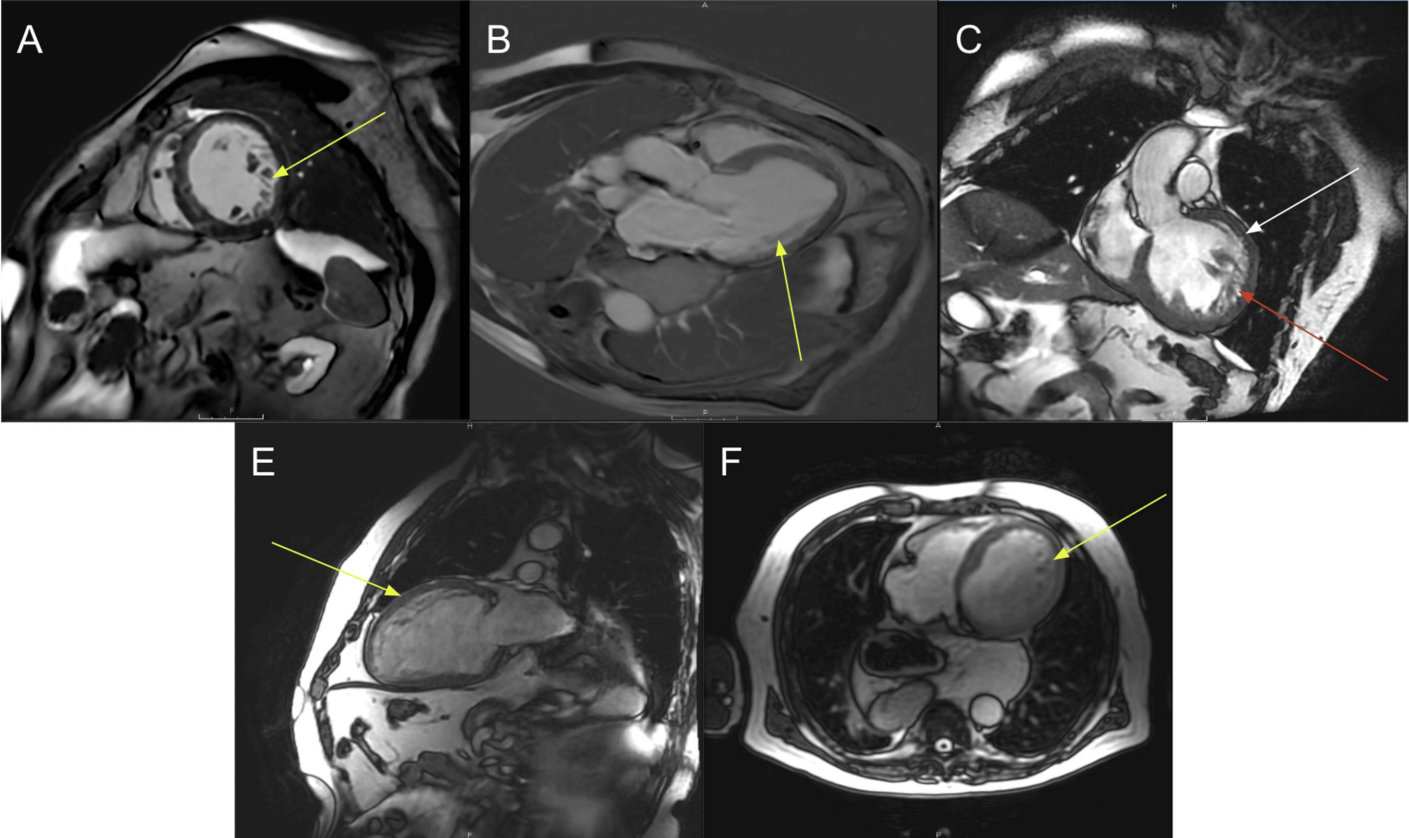
Cardiac MRI (A) Sagittal. (B) Short-axis apical. (C) Three-chamber vertical long axis. (D) Coronal. (E) Short-axis mid-diastole. (F) Axial. Cardiac MRI images reveal an increased trabeculated-to-non-compact myocardium in the left ventricle (yellow arrows). The ratio of non-compacted (white arrow) to compacted myocardium (red arrow) is 2.5.

**Figure 3:**
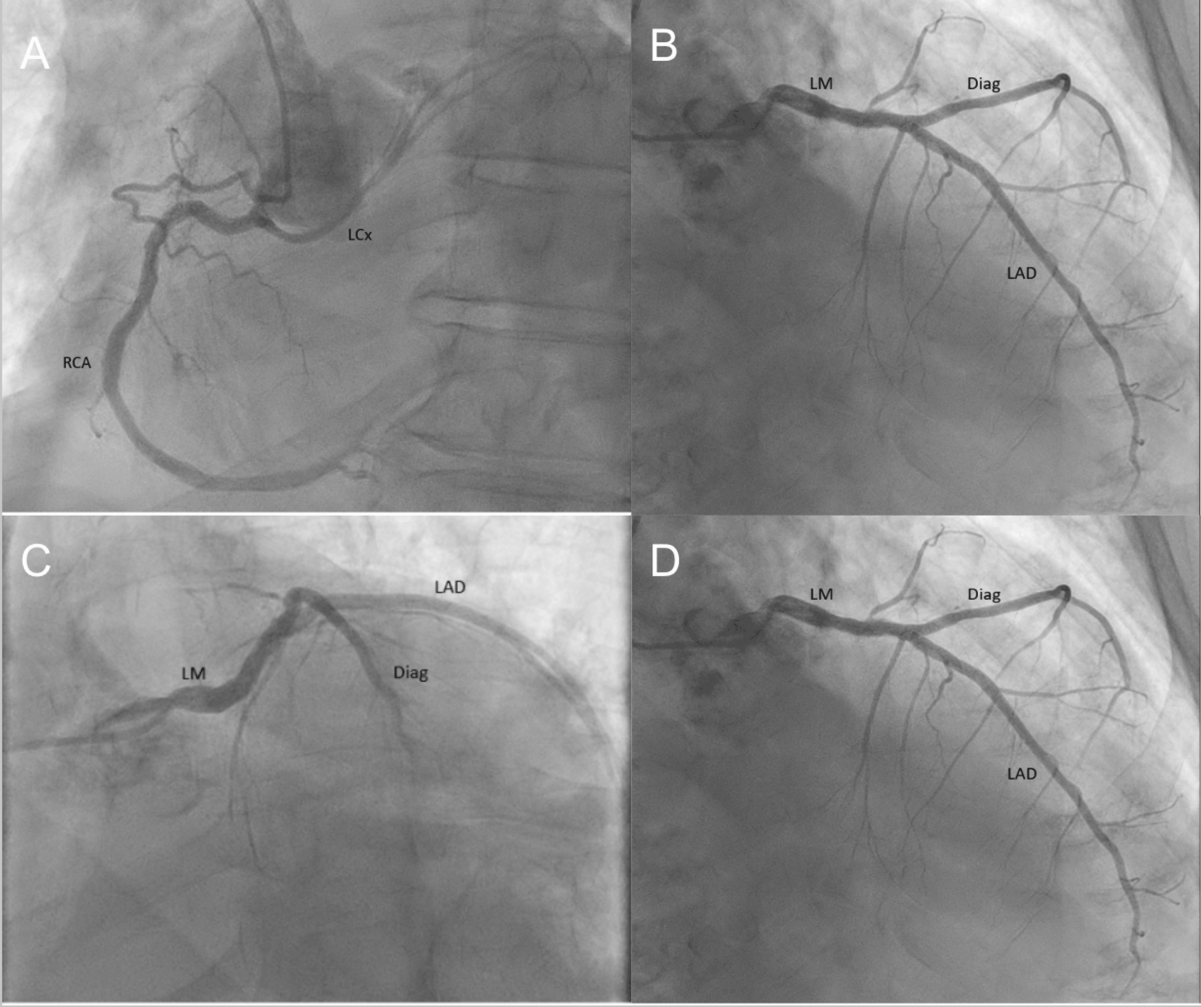
Coronary angiography showing an anomalous left circumflex artery The LCX originates anomalously from the proximal RCA. It is a large-caliber but non-dominant vessel (A), giving rise to a medium-caliber first obtuse marginal branch, which further bifurcates in its proximal segment. The LM branches into the LAD and the Diag (A, B, C, D). Diag, diagonal artery; LAD, left anterior descending artery; LCX, left circumflex artery; LM, left main coronary artery; RCA, right coronary artery

A follow-up cardiac MRI performed during the admission confirmed extensive left ventricular trabeculations and quantified a noncompacted-to-compacted myocardial thickness ratio exceeding 2.3 in diastole (Figure 2), meeting the accepted diagnostic criteria for LVNC. Subsequent right and left heart catheterization revealed nonobstructive epicardial coronary artery disease, an anomalous left circumflex artery originating from the proximal right coronary artery (Figure 3), and elevated filling pressures, including a pulmonary artery systolic pressure of 50 mmHg. Given his severely reduced EF (<20%), low cardiac output (1.6 L/min), and index (0.8 L/min/m²), along with markedly elevated right-sided pressures, his presentation was consistent with an advanced stage of heart failure.

Therapeutically, he received an amiodarone infusion for rate control of atrial fibrillation, along with his outpatient anticoagulation regimen (dabigatran). A transesophageal echocardiogram-guided cardioversion successfully restored normal sinus rhythm. Maintenance therapy included amiodarone (200 mg daily) and digoxin (125 mcg daily), in addition to sacubitril/valsartan (24/26 mg twice daily) and metoprolol succinate (25 mg daily) as guideline-directed medical therapy (GDMT) for heart failure with reduced EF. Given his profound left ventricular dysfunction, an ICD was planned for primary prevention of sudden cardiac death.

Although the new diagnosis of LVNC did not substantially alter the core management of systolic heart failure (i.e., GDMT), it emphasized the need for rigorous follow-up and device-based therapy. His case most likely represented a sporadic (nonfamilial) form of LVNC, given the late age at diagnosis, absence of known familial cardiomyopathy, and lack of LVNC features in first-degree relatives. If familial disease had been suspected, screening echocardiography for relatives would have been recommended. However, the patient’s advanced age and progressive structural changes - likely exacerbated by longstanding hypertension, atrial fibrillation, and a chronic cardiomyopathy of undetermined origin - support the concept of LVNC arising from adverse myocardial remodeling rather than a purely congenital or hereditary etiology.

## Discussion

LVNC is increasingly recognized as a heterogeneous cardiomyopathy influenced by both genetic and acquired factors [1,2]. While embryological arrest in myocardial compaction has long been implicated, conditions such as longstanding hypertension or myocarditis may also contribute to adverse remodeling, leading to a noncompacted phenotype [2,3]. Genetic testing in LVNC frequently identifies mutations in genes such as *MYH7*, *TTN*, and *LMNA*, with early detection aiding in family screening and targeted interventions [1,4]. However, diagnosing LVNC remains challenging due to its overlapping features with dilated and hypertrophic cardiomyopathies, as well as evolving diagnostic criteria, including noncompacted-to-compacted ratio thresholds [2,5].

Patients exhibit varying degrees of heart failure symptoms, arrhythmias, and thromboembolic complications, posing therapeutic challenges. Arrhythmia management is particularly complex given the increased risk of ventricular tachyarrhythmias and syncope, alongside the progression of systolic dysfunction and the need for sudden cardiac death risk stratification. In this case, the patient’s severely reduced EF (<20%) and significant arrhythmic burden supported the decision to proceed with ICD placement [6,7]. Although atrial fibrillation was the primary contributor to the patient’s stroke risk, the morphological complexity of LVNC may also predispose non-atrial fibrillation patients to intracardiac thrombus, raising questions about prophylactic anticoagulation despite the absence of definitive guidelines [3]. Furthermore, the presence or absence of late gadolinium enhancement on MRI offers additional prognostic insights and may help guide advanced therapy decisions. While emerging approaches such as gene therapy, novel imaging techniques, and left ventricular assist devices hold promise, their optimal integration into LVNC management remains uncertain [5,8,9].

Although coronary angiography in this patient revealed an anomalous origin of the left circumflex artery, this structural anomaly - one of the more commonly encountered coronary variants - did not significantly impact ischemic risk or require specific intervention, making it largely incidental. Nonetheless, certain coronary anomalies can complicate LVNC management by introducing additional ischemic considerations or affecting surgical planning. In this case, recognizing that the patient’s preexisting cardiomyopathy and atrial fibrillation warranted further evaluation ultimately led to the correct diagnosis of LVNC via echocardiography and cardiac MRI. If the condition had been familial, formal screening of relatives would have been recommended; however, the patient’s late age at diagnosis and lack of family history suggest a sporadic presentation. These findings underscore the critical role of multimodality imaging, comprehensive arrhythmia management, and individualized heart failure therapies in optimizing outcomes for LVNC patients, while also highlighting areas of ongoing investigation, such as prophylactic anticoagulation, ICD criteria for EFs above 35%, and the role of advanced therapies.

## Conclusions

In this case, an older adult with chronic atrial fibrillation and preexisting nonischemic cardiomyopathy was diagnosed with LVNC, highlighting the potential for a later-life, nonfamilial presentation likely influenced by adverse myocardial remodeling. Multimodality imaging, including echocardiography, cardiac MRI, and coronary angiography, was crucial not only for confirming the diagnosis and assessing noncompaction severity but also for identifying coexisting anomalies such as the anomalous left circumflex artery. These findings emphasize the importance of comprehensive cardiac evaluation in patients with atypical or progressive heart failure, particularly in the presence of arrhythmias, and reinforce the value of guideline-directed pharmacotherapy, rate or rhythm control, and ICD placement in reducing the risk of sudden cardiac death.

Despite the insights gained from this case, important challenges remain. LVNC’s highly variable presentation continues to blur its diagnostic boundaries, leading to underdiagnosis, particularly in older populations. Further large-scale studies are needed to refine imaging criteria, clarify indications for prophylactic anticoagulation, especially in non-atrial fibrillation patients, and explore the role of genetic testing and emerging therapies in guiding more personalized care. Addressing these gaps will help optimize treatment strategies, improve risk assessment, and enhance long-term outcomes for individuals with this rare but clinically significant cardiomyopathy.
